# Posterior cruciate ligament balancing in total knee arthroplasty: a numerical study with a dynamic force controlled knee model

**DOI:** 10.1186/1475-925X-13-91

**Published:** 2014-07-02

**Authors:** Arnd Steinbrück, Matthias Woiczinski, Patrick Weber, Peter Ernst Müller, Volkmar Jansson, Christian Schröder

**Affiliations:** 1 Department of Orthopedic Surgery, Physical Medicine and Rehabilitation, University Hospital of Munich (LMU), Campus Großhadern, Marchioninistraße 15, 81377 Munich, Germany

**Keywords:** Finite element model, Ligament balancing, TKA, Knee kinematics, Posterior cruciate ligament

## Abstract

**Background:**

Adequate soft tissue balancing is a key factor for a successful result after total knee arthroplasty (TKA). Posterior cruciate ligament (PCL) is the primary restraint to posterior translation of the tibia after cruciate retaining TKA and is also responsible for the amount of joint compression. However, it is complex to quantify the amount of ligament release with its effects on load bearing and kinematics in TKA and limited both *in vivo* and *in vitro*. The goal of this study was to create a dynamic and deformable finite element model of a full leg and analyze a stepwise release of the PCL regarding knee kinematics, pressure distribution and ligament stresses.

**Methods:**

A dynamic finite element model was developed in Ansys V14.0 based on boundary conditions of an existing knee rig. A cruciate retraining knee prosthesis was virtually implanted. Ligament and muscle structures were simulated with modified spring elements. Linear elastic materials were defined for femoral component, inlay and patella cartilage. A restart algorithm was developed and implemented into the finite element simulation to hold the ground reaction force constant by adapting quadriceps force. After simulating the unreleased PCL model, two models were developed and calculated with the same boundary conditions with a 50% and 75% release of the PCL stiffness.

**Results:**

From the beginning of the simulation to approximately 35° of flexion, tibia moves posterior related to the femur and with higher flexion anteriorly. Anterior translation of the tibia ranged from 5.8 mm for unreleased PCL to 3.7 mm for 75% PCL release (4.9 mm 50% release).

A decrease of maximum von Mises equivalent stress on the inlay was given with PCL release, especially in higher flexion angles from 11.1 MPa for unreleased PCL to 8.9 MPa for 50% release of the PCL and 7.8 MPa for 75% release.

**Conclusions:**

Our study showed that dynamic FEM is an effective method for simulation of PCL balancing in knee arthroplasty. A tight PCL led in silico to more anterior tibia translation, a higher collateral ligament and inlay stress, while retropatellar pressure remained unchanged. Surgeons may take these results in vivo into account.

## Introduction

A relevant key factor for a successful TKA is a balanced flexion and extension gap to allow normal knee kinematics and loading patterns of the knee joint [1]. The PCL is in cruciate retaining knee arthroplasty systems one main factor to control anterior-posterior translation [2-4] and is also responsible for the amount of joint compression [5]. However, to quantify the amount of ligament release and its effect on load bearing and kinematics in TKA is complex and limited both *in vivo* and *in vitro*. Knee cadaver studies are a possibility to investigate such issues, but they are complex, cost- and time-intensive [6]. Furthermore, there are difficulties in exact measuring stress and strain distributions or knee kinematics and certain limitations, such as hardly any possibility to vary ligament stiffness in the identical cadaver.

Computer simulations with finite element models are an efficient and common way to study knee biomechanics [7-16]. Some computational knee models were static or quasi static and focused on femoral component alignment [12], patellofemoral contact pressure distributions [9] or quantification of soft tissue balance in TKA [13,16]. Dynamic knee models were also developed and studied for joint laxity [15] or kinematics during gait cycle [10]. Some of these studies were based on boundary conditions of mechanical simulators or knee rigs [7,10,11] and a few of these models were based on a full leg including hip and ankle joint [7,13-15].

The goal of this study was to create a dynamic and deformable finite element model of a full leg with six degrees of freedom (DOF) to investigate clinical relevant questions on the human knee joint. It was therefore required that the model should be force controlled to hold a constant ground reaction force. The model in this study should be capable to investigate four hypotheses of PCL balancing after TKA during a weight bearing squat: (1) There is an influence on knee kinematics by releasing PCL. (2) There is an altered stress distribution on the inlay with different PCL stiffness. (3) There are differences on retropatellar peak pressure or distribution by changing PCL stiffness. (4) Collateral ligaments stresses are influenced by a PCL release.

## Materials and methods

### 3D-model generation

A finite element model of a full left leg was created for a dynamic force controlled computer analysis in Ansys V14.0 (Ansys, Inc., Canonsburg, PA, USA).

Therefore, Magnetic Resonance Image (MRI) data of a full leg were used to develop the finite element simulation within correct anatomical circumstances. A male (28 years, 80 kg, 173 cm) control subject was scanned for this study. The knee was scanned in 15° of knee flexion. The MRI data consisted of slices with a separation distance of 1 mm in the sagittal plane. There were no signs of surgical intervention or pathologic condition related to the lower extremity on the examined knee. The contours of femur, tibia, fibula, patella as well as the articular cartilage of the knee were segmented manually by an engineer with the help of a radiologist and and an orthopaedic surgeon using the software package Amira (Visage Imaging GmbH, Berlin, Germany). For an accurate and more compact dataset the models were converted into non-uniform rational b-splines (NURBS) surface by the reverse engineering software Geomagic Studio (Geomagic Inc., Research Triangle Park, Morrisville, NC, USA).

### Preparation and virtual implantation of the prosthesis

The computer aided design data of the fixed bearing total knee prosthesis Columbus (Aesculap Orthopaedics, Tuttlingen, Germany) were provided by the manufacturer. To guarantee a good mesh quality, cement pockets on the tibia and femoral component, the coupling mechanism for revision and the femoral condyles pins were removed using CAD-software (Catia V15R19 Dessault Systems HQ,Velizy-Villacoublay, France).

In a next step the 3D-models of the knee and the total knee prosthesis were imported in the implicit software module of the finite element software Ansys V14.0. The virtual implantation of the prosthesis was done with the geometry modeler module, which is included in the Ansys software package. The prosthesis was implanted according to the recommendations of the manufacturer, in a neutral position, aligned perpendicular to the mechanical axis of the knee. Rotation for the femoral component was zero degrees to the anatomical transepicondylar axis. The internal/external rotation of the tibia plateau was aligned to the center of the distal tibia. Tibial cut was aligned with zero degrees to the proximal anatomical axis [17], while a posterior slope of 3 degrees is included in the inlay design.

The size and alignment of the implant were examined by an experienced orthopaedic surgeon to ensure correct implantation.

### Mesh generation and convergence analyses

For mesh generation, quadratic 10-node tetrahedral elements were used for articular cartilage and the polyethylene (PE) inlay. The femoral component in the model was assumed to be rigid and only the surface was meshed.

A convergence analysis with mesh sizes from 3 mm down to 1.5 mm was performed. In the present study, the equivalent stress on patella and inlay and furthermore the maximum error energy were considered as convergence criteria. The error energy within each element was calculated as

(1) $$e_{i}=\frac{1}{2}\int_{\vartheta }{\left\{\Delta \sigma \right\}}^{T}{\left[D\right]}^{-1}\left\{\Delta \sigma \right\}d\vartheta \;$$

where *e*_*i*_ was the error energy in element i, {*Δσ*} was the nodal stress error,  *ϑ* was the volume of the element, and {*D*} was the stress strain matrix. The nodal stress error {*Δσ*} was the averaged nodal stresses minus the unaveraged nodal stresses [18].

### Material properties and contact conditions

Linear elastic material properties were defined for the prosthesis after they had been verified in a former study [19]. Also, the unresurfaced patella cartilage was considered to behave linear elastically which was described by Pena et al. [14] (Table 1A). Ligament structures were simulated with a Link180 spring element, which allows adding a pre-strain and supports force in tension only. Lateral collateral ligament (LCL) was simulated with one bundle, medial collateral ligament (MCL) with three bundles (anterior (MCLa), oblique (MCLo) and deep bundle (MCLd)) and the posterior cruciate ligament (PCL) with two bundles (anterior (PCLa) and posterior bundle (PCLp)). Reference strains and stiffness were assigned to the ligaments based on previous finite element models on the knee (Table 1B) [20]. Correct positions of the ligaments were checked by anatomical landmarks based on former dissection studies of the human knee [21-23].

**Table 1:** **A) Linear elastic material properties of model components **[19]** B) Ligament properties **[20]

***A***	**Component**	**Young’s modulus (MPa)**	**Poisson ratio**
	Femoral component (CoCr)	217000	0.3
	Inlay (PE)	312.5	0.46
	Articular cartilage	5.0	0.46
***B***	**Ligament**	**Stiffness (N/mm)**	**Reference strain at 15° of flexion**
	LCL	91.3	0.02
	MCL anterior	27.9	0.02
	MCL oblique	21.1	0.02
	MCL deep	72.2	0.02
	PCL anterior	125.0	−0.10
	PCL posterior	60.0	−0.02

Frictional contacts were added with a frictional coefficient of μ = 0.05 for the femorotibial and μ = 0.02 for the patellofemoral joint based on results from other studies [24-26].

### Boundary conditions and muscles

**Figure 1:**
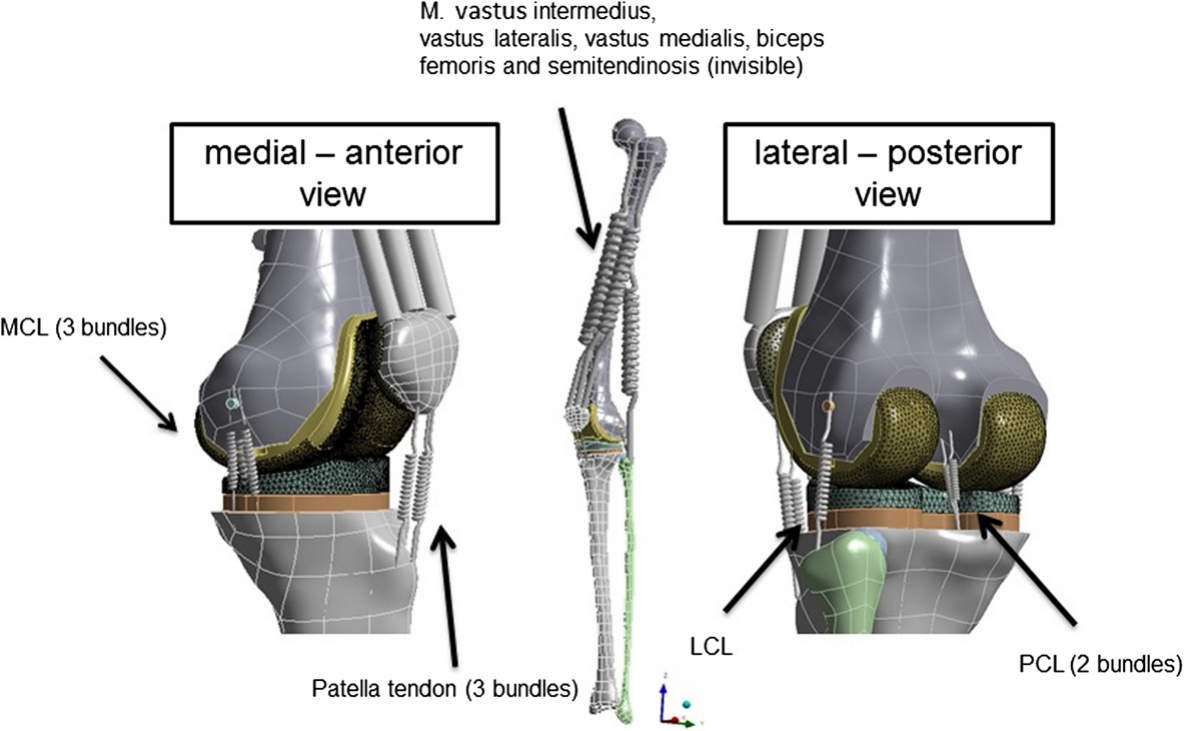
**Boundary conditions, muscles and ligaments in the finite element simulation.** Medial collateral ligament (MCL), Lateral collateral ligament (LCL) and Posterior cruciate ligament (PCL).

The boundary conditions of the finite element model were conducted in relation to an existing knee rig [27]. The origin of the reference coordinate system was on the ankle joint (Figure 1). All translations on tibial side were prohibited and all rotations were free (3 degrees of freedom (DOF)). The femoral head was supported with a remote displacement in z-direction (4th DOF). Additionally, the rotation of the x-axis (5th DOF; flexion/extension) and y–axis (6th DOF; varus/valgus) were free.

To simulate a dynamic squad, 30 load steps were added to the simulation and the center of the femoral head moves during the simulation on the z-axis from 0 mm displacement to 150 mm to the ankle joint. All other bodies had no boundary conditions to ensure a free moving knee model with 6 DOF.

Vastus lateralis, vastus medialis were simulated with a constant load of 20 N and hamstring muscles (biceps femoris and semitendinosis) were simulated with a constant load of 2 × 10 N during the whole flexion cycle.

**Figure 2:**
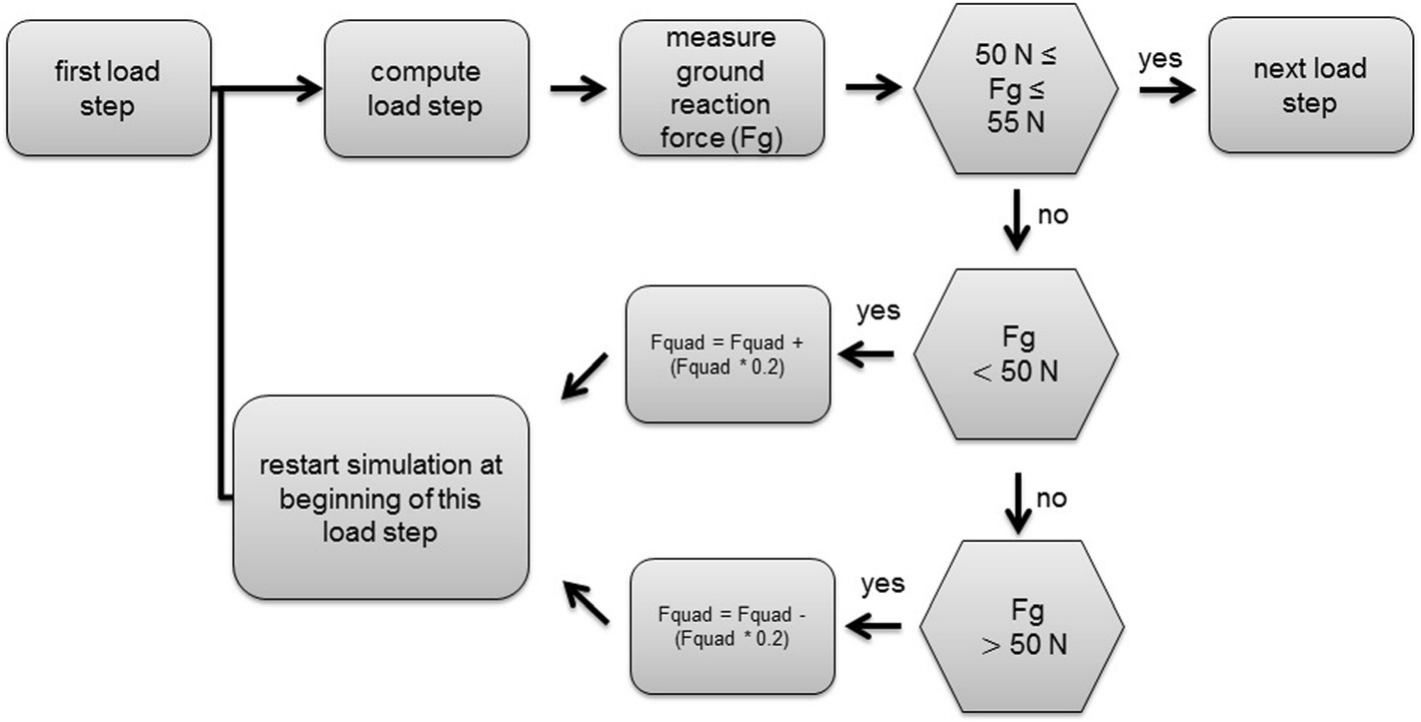
**Algorithm for restart procedure to ensure a constant ground reaction force.** Ground reaction force (Fg); Quadriceps force (Fquad).

To ensure the alignment of the muscles during flexion cycle, spring elements were added to the simulation and reprogrammed using program language Ansys parametric design language (APDL). The adaption of the muscle elements for vastus medialis, vastus lateralis and hamstring muscles included a constant preload of the spring element. A very small stiffness of 0.001 N/mm ensured a constant preload over the whole simulation due to changing length of the spring because of the geometrical circumstances during flexion.Vastus intermedius muscle was also simulated with a spring element. However, further adaption to this element was necessary to generate a dynamically controlled, fully automated finite element model with a constant ground reaction force. During knee flexion the reaction force of the ankle joint (z-direction) was measured at each load step. If the ankle force was not between 50 N and 55 N, the load step restarted with an adapted load of vastus intermedius muscle until the predefined force range was reached (Figure 2).

### Different model setups for PCL balancing

Three finite element models based on the description above were generated to evaluate results on knee kinematics and stress distribution due to changes in PCL stiffness. In each model set-up the PCL stiffness was changed. First the initial model with a tight PCL (no release, reference strain at 15° of flexion, see Table 1B) was created and calculated. Afterwards a PCL release on both bundles of 50% and 75% was performed and these models were analyzed respectively.

### Statistics

For analyzing the effect of a PCL release on knee kinematics and -/or mechanical stress changes in prosthesis components the following data were analyzed during the whole range of motion by descriptive statistics:

– force of quadriceps muscle

– anterior-posterior translation of the tibia measured in the central in relation to the epicondyles of the femur

– von Mises equivalent stress on patella cartilage and inlay

– load curves of collateral ligaments (LCL, MCLa, MCLo and MCLd)

## Results

**Figure 3:**
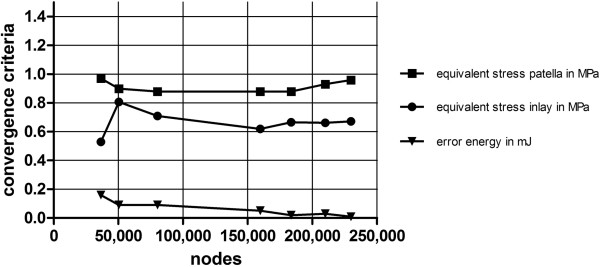
Results of the convergence study of the mesh.

Convergence analysis showed homogeneous results with a minimum mesh size of 1.5 mm. Finally, the model had in total 123,462 elements and 229,812 nodes for all different PCL set-ups (Figure 3).

**Figure 4:**
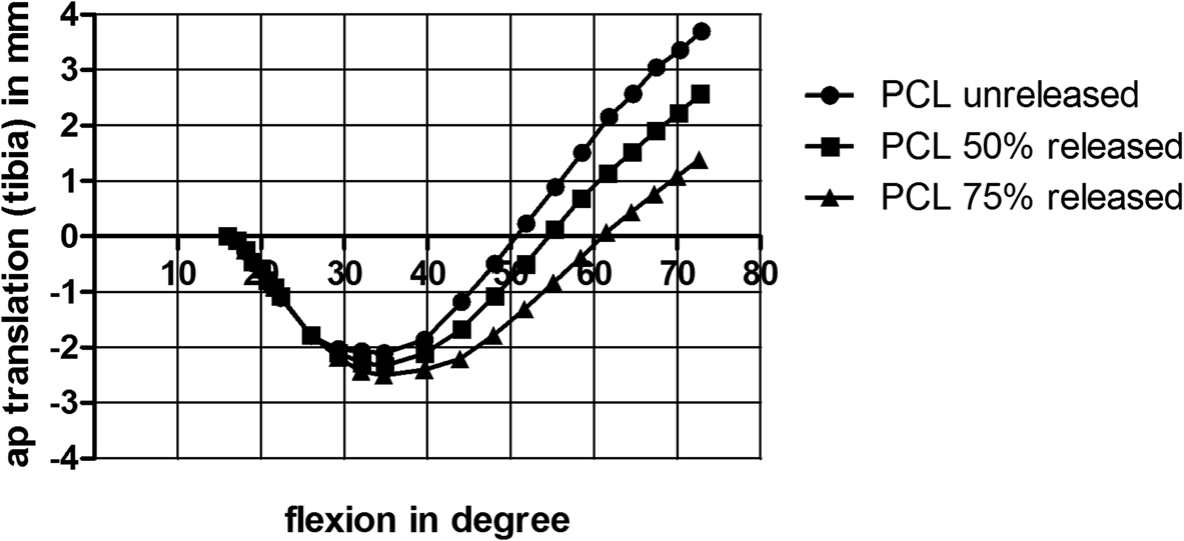
Anterior-posterior translation of tibia related to femur: positive tibia moves anterior; negative tibia moves posterior.

**Figure 5:**
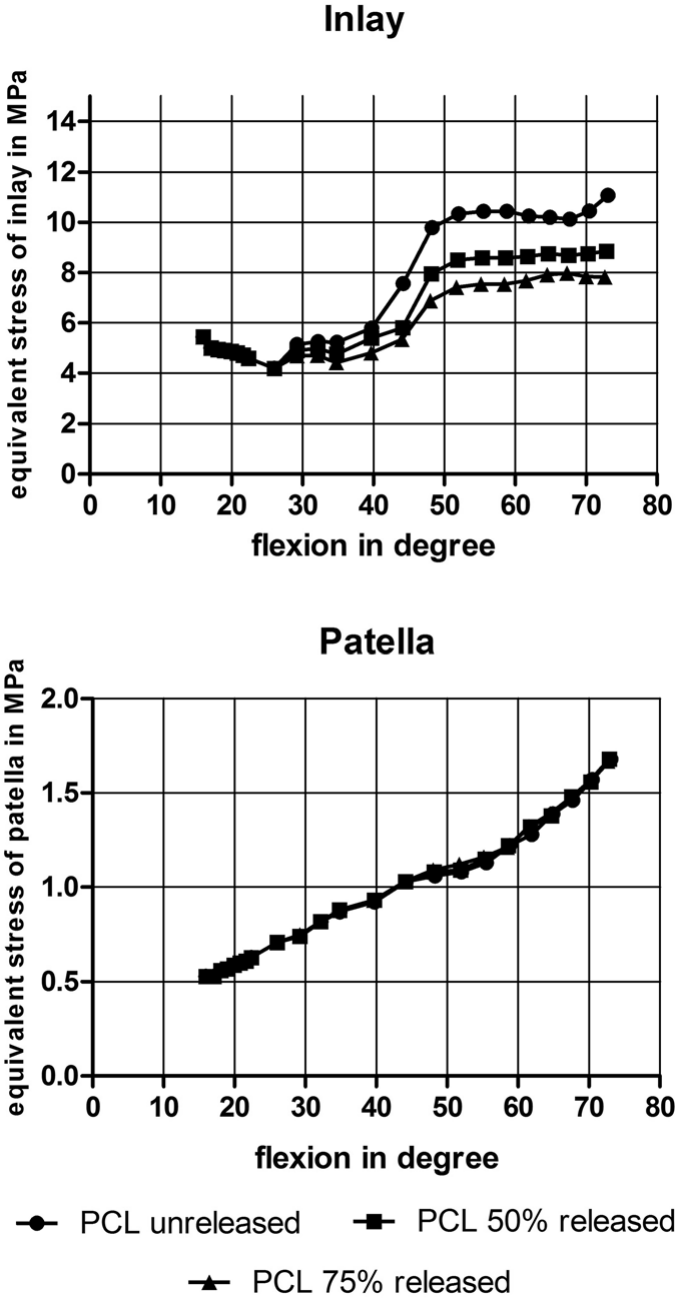
Von Mises equivalent stress on inlay (top) and Patella cartilage (bottom) of the different models in MPa.

**Figure 6:**
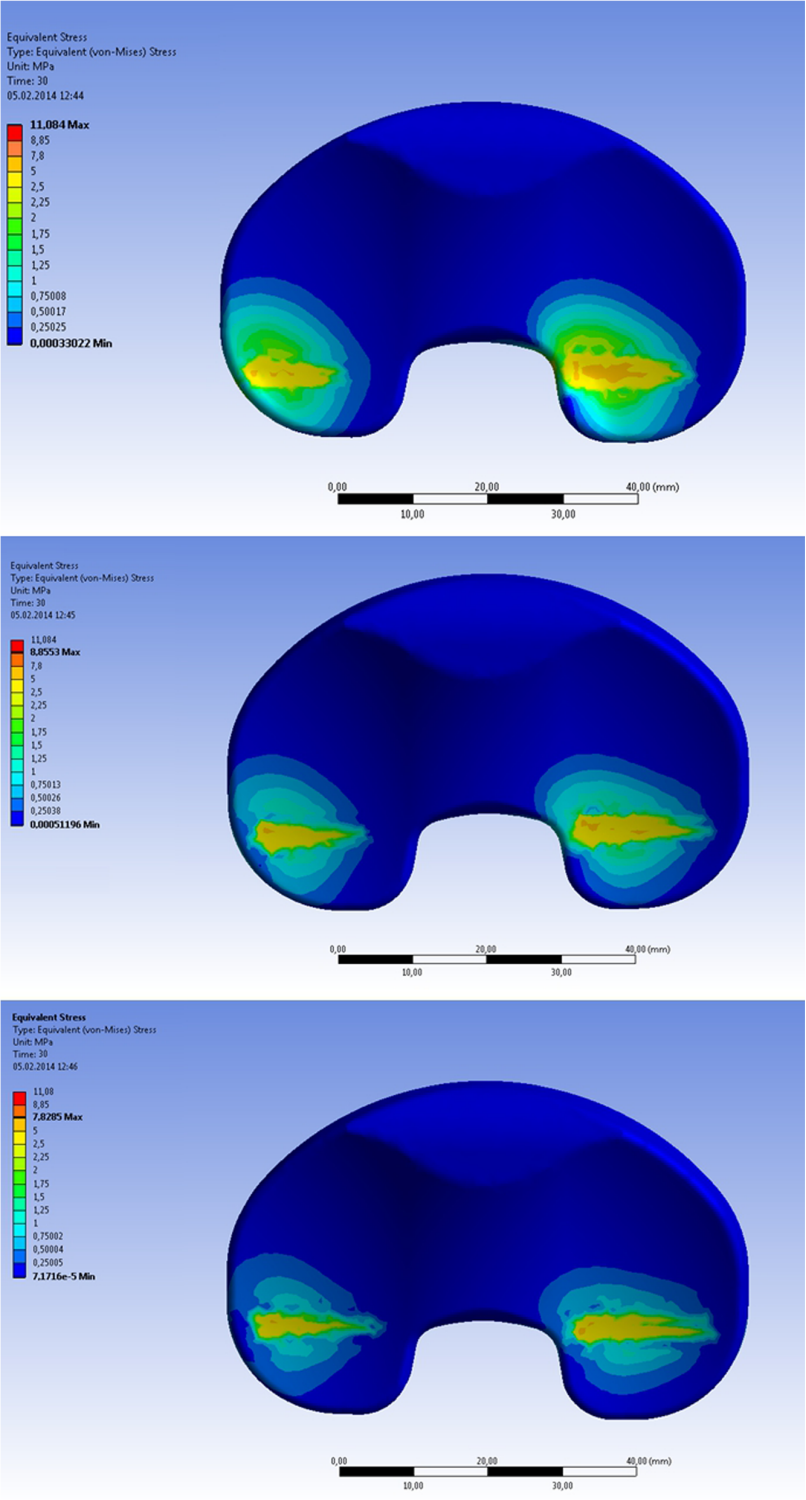
**Equivalent stress on the inlay in MPa at full flexion.** Top: PCL unreleased; Middle: PCL 50% released; Bottom: PCL 75% released.

All three different model setups remained stable through the range of motion of 15.98° + 0.01° to 72.66° ± 0.42° and the ground reaction force of each model was in the required range between 50 N and 55 N at each degrees of flexion. Solution time for each model was up to 24 hours on a workstation with two Intel Xeon (2.4 GHZ) and 12 GB RAM. All three models had no changes in quadriceps force: For unreleased PCL, 50% and 75% PCL release quadriceps force was 21.18 N  0.01 N at initial flexion and 403.43 N 1.89 N at full flexion.The center of the tibia translated posterior in relation to the femur from the beginning of the squad until 35° of flexion and then anteriorly until the end of the flexion. From 16° to 35° of flexion the PCL 75% release model had a posterior movement of 2.5 mm, the PCL 50% release model had a posterior movement of 2.3 mm and the PCL unreleased model had a posterior movement of 2.1 mm. From 35° of flexion to the end of the simulation maximal movement from posterior to anterior ranged from 5.8 mm for unchanged PCL to 3.7 mm for 75% PCL release. For PCL 50% release maximal anterior movement of the tibia was 4.9 mm. The position of the tibia in the maximum flexed position was 1.4 mm more anterior than the initial position for PCL 75% release and 3.7 mm more anterior for PCL unreleased. For PCL 50% release tibia was 2.6 mm more anterior than in the initial position (Figure 4). The PCL unreleased model had in maximum 1.8° internal rotation and 0.5° more internal rotation at 61° of flexion than the PCL 50% release model and 0.8° more internal rotation than the PCL 75% release model.Von Mises equivalent stress on the inlay had an initial maximum value of 5.45 MPa in each PCL set-up at beginning and had no changes before a flexion of 28° of flexion. From 28° to 40° of flexion the maximum of von Mises equivalent stress decreased from 5.7 MPa on the inlay for unreleased PCL to 5.4 MPa for 50% release and to 4.8 MPa for 75% release of the PCL. Furthermore, a decrease of the maximum von Mises equivalent stress was given with PCL release in higher flexion angles (40° to 73° of flexion) from 11.1 MPa for unreleased PCL to 8.9 MPa for 50% release of the PCL and 7.8 MPa for 75% release of the PCL (Figures 5 and 6).

**Figure 7:**
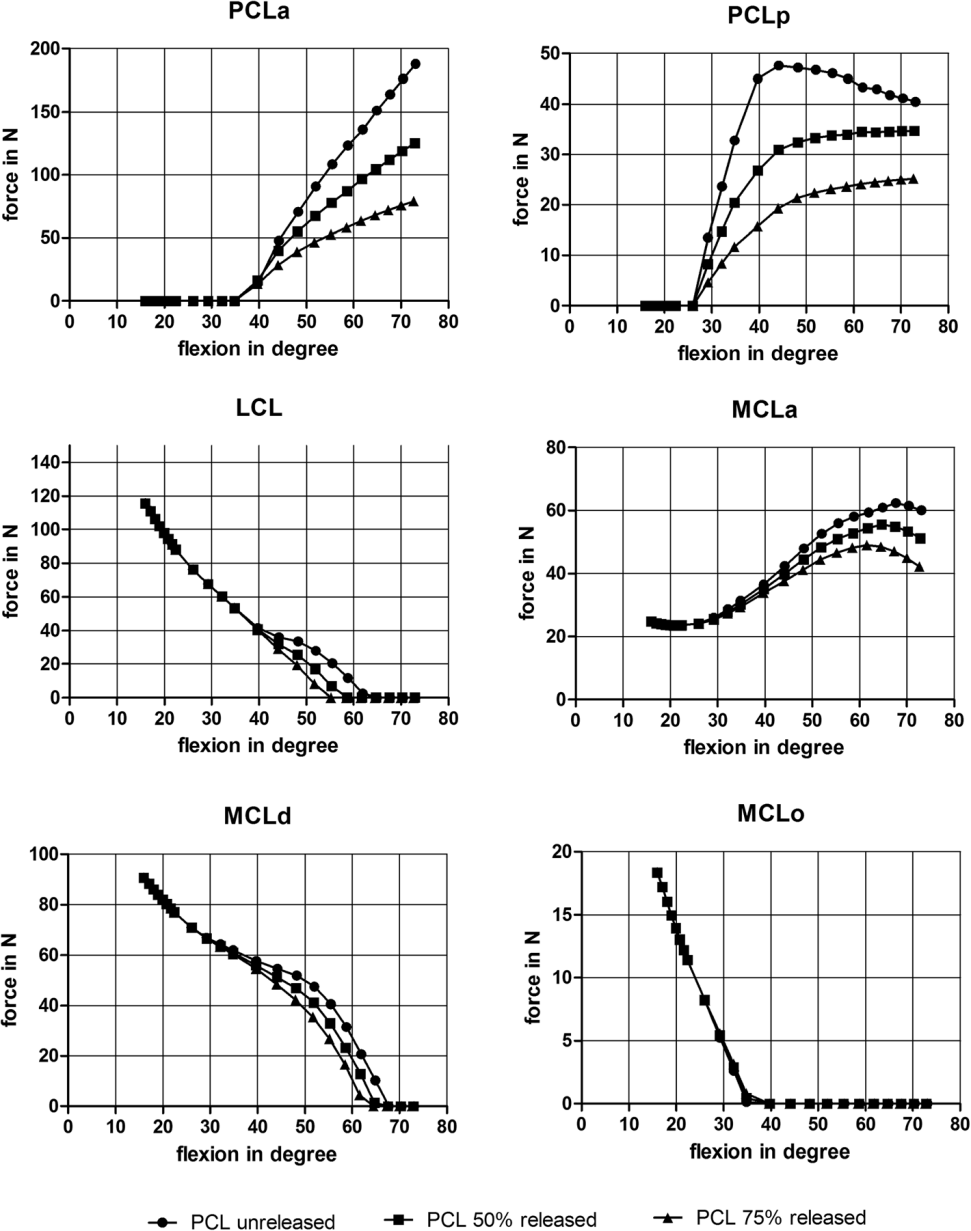
**Ligament force during the whole flexion cycle.** Medial collateral ligament (MCL), Lateral collateral ligament (LCL) and Posterior cruciate ligament (PCL); Anterior bundle = a; posterior bundle = p; deep bundle = d; oblique bundle = o.

Quadriceps force and retropatellar pressure increased continuously with the flexion of the knee. Maximum quadriceps force at full flexion was 404 N ± 2 N. Maximum equivalent stress of each model was 0.53 MPa at the beginning of the simulation and 1.67 MPa  0.01 MPa at full flexion. Although there was no change of von Mises equivalent stress on the patella cartilage for the three different PCL release models (Figure 5).PCLa was (relatively) slack until 35° of flexion and PCLp until 26° of flexion. From 26° to 73° of flexion both ligaments were more loaded in PCL unreleased then in PCL 50% release and PCL 75% release. No changes in load were seen in LCL ligament for PCL release before a flexion of 40° of flexion. From 40° to 62° of flexion LCL had slightly more load in the unreleased PCL model (27.8 N at 52° of flexion) than in the 50% (19.9 N at 52° of flexion) and the 75% PCL release model (8.2 N at 52° of flexion). After 62° of flexion the LCL ligament was (relatively) slack again. MCL bundels changed differently through the range of motion for PCL release. For MCLa there was no change in load until 29° of flexion (26 N).The unreleased PCL model had more load on MCLa (60 N at 73° of flexion) than the PCL 50% release model (51 N at 73° of flexion) and the PCL 75% release model (42 N at 73° of flexion). The slope of the ligament load curve during flexion was higher for the unreleased PCL model then for the PCL 50% release and 75% release model (Figure 7). The MCLd load did not change from 16° to 30° of flexion. In the range of 30° to 68° of flexion, the unreleased PCL model had a higher load (40 N at 55° of flexion) then the PCL 50% release (33 N at 55° of flexion) and 75% release model (26 N at 55° of flexion). After 68° of flexion, the MCLd bundle experienced no load. The MCLo bundle had almost no changes during the whole range of motion (Figure 7).

## Discussion

Adequate soft tissue balancing is one of the key factors for a successful result after TKA [1]. Inadequate soft tissue tension may cause an unbalanced flexion and extension gap leading to instability and a higher revision rate [28,29]. PCL is the primary restraint to posterior translation of the tibia related to the femur after cruciate retaining TKA [2,4]. Heesterbeek et al. showed in their intraoperative study, that if the PCL is tight a tibial anterior subluxation can occur and femoral component condyles bear on the posterior edge of the PE inlay [5]. This can lead to limited flexion and higher contact stresses on PE inlay [30]. A tight PCL might intra-operatively be recognized by the “open-book kinematics” with tibial tray lift-off in flexion during trial implantation [30,31].

However, to quantify the amount of a ligament release and its effect on load bearing and kinematics of the knee is complex. Finite element models in knee biomechanics are now a common way to investigate different clinical or biomechanical issues [7-16,20]. Barink et al. stated that modeling the bones and ligaments of the knee from MRI scans may allow a level of control on loading and boundary conditions impossible with *in vivo* or *in vitro* testing, and avoids ethical issues inherent with testing on tissues [15]. There are a few numerical model studies which are investigating ligament balancing of the knee [13,15,16]. Oh et al. [13] did not include a dynamic simulation, therefore results are limited to the standing phase of the knee and no kinematic or mechanical observation during flexion is possible. Our study demonstrated that it is possible to simulate a knee bend with a dynamic force controlled finite element model with natural circumstances of a full leg to 73° of flexion. The study by Barink et al. [15] was dynamic but avoided to include the increasing quadriceps muscle force during knee bend. For a dynamic knee bend solution which should be possible to evaluate results during the whole range of motion the changing quadriceps muscle force is a key factor. Avoiding a changing in quadriceps muscle force maybe ending in faster computational solution time, but if there is no change in quadriceps muscle force during the range of motion all kinematic and mechanical changes are not showing the right conditions for a real knee bend. Hence, our study demonstrated that with a programmed restarting procedure in the numerical solution it is possible to simulate a more realistic knee bend but resulting in computation time up to 24 hours on a fast workstation (two Intel Xeon @2.4 GHZ with 12 GB ram).

Our study gives evidence that in case of an tight PCL a release may lead to a relevant decrease of anterior translation of the tibia, which is in accordance with clinical studies [5]. Varadarajan et al. showed in his review of seven in vivo and in vitro studies an anterior tibia translation during flexion for cruciate retaining knees to a maximum of 10 mm at 90° of flexion, but also showed in some studies nearly no ap translation. In our study the knee bend stops at 70° of flexion this might be a reason for less ap translation of 5.8 mm of our unreleased model. Tibial internal rotations were slightly lower by PCL releasing which is in good accordance to Li et al. who showed similar findings in low flexion angles until 60° of flexion [32].

We also showed a relevant decrease of the joint compression after releasing the PCL from 11.08 MPa to 7.83 MPa for 75% release of the PCL. The amount of joint compression seems to be similar for FE studies, if the difference in ground reaction force is taken into account [16]. Zelle et al. [16] for example showed a maximum equivalent stress of 26 MPa on the inlay, but simulated a ground reaction force of 350 N. In our study a ground reaction force of 50 N was simulated which could be an explanation for the generally lower equivalent stress on the inlay [16]. Furthermore, our study showed that a tight PCL leads to a higher stress on the inlay and more anterior-posterior movement. These two factors might increase the polyethylene wear in knee prostheses [33,34]. Even collateral ligament stress was diminished with an increasing release of the PCL from certain degree of flexion, which can be explained with less anterior translation of the tibia.

While retropatellar pressure increased with flexion of the knee, releasing of the PCL had no effect on the retropatellar contact patterns. Equally quadriceps force did not alter relevantly after PCL release, which may also explain the constant retropatellar peak pressure in different PCL releases.

Müller et al. showed in their study a changing knee kinematic with increasing ankle force but also stated that the profiles remained similar. This indicates that clinical questions can be elucidated with partially loaded knees and an increasing ankle force not guarantees better results. In our study an ankle force of 50 N was used and therefore the results may be transmittable to full weight bearing knees [35]. A limitation of our study was that we did not analyze higher flexion grades of the knee. The simulation was manually stopped at 73° of flexion to avoid quadriceps tendon contact to the femur, because its contact was not included in our numerical model. Although knee flexion until 70° is specific for normal walking [36] and our results may be transmitted to this daily activity. Victor et al. showed in an in vitro setup that significant differences in knee kinematics existed between different muscle actions in the knee during squatting. For example there is a difference in knee kinematics between the simulations with the quadriceps muscle only and together with hamstrings activation. Therefore in this investigation hamstring muscles were simulated, although it is a limitation of the study, that not all muscles of the knee were simulated. The ligaments were not fully reconstructed as meshed bodies and material properties used in the whole simulation were linear elastic. Ligaments were reconstructed with spring elements in bundle technique, which is a common technique in finite element studies and showed good agreement with experimental setups [7,20].

## Conclusions

Despite the described limitations, our study demonstrated that FEM is an elegant method for simulation of posterior cruciate ligament balancing in knee arthroplasty. A tight PCL may alter the knee kinematics with more anterior tibial translation with flexion. Furthermore a stiffer PCL leads to a higher collateral ligament and inlay stress. Although retropatellar pressure remains unchanged under different PCL conditions. Surgeons could take these results in account intra-operatively. Further studies should include higher flexion grades of the knee and simulation of daily activities.

## Abbreviations

APDL: Ansys parametic design language; CoCr: Cobalt chromium; DOF: Degrees of freedom; FEM: Finite element method; Fg: Ground reaction force; Fquad: Quadricepsforce; LCL: Lateral collateral ligament; MCLa: Medial collateral ligament anterior bundle; MCLd: Medial collateral ligament deep bundle; mm: Millimeter; MPa: Mega Pascal; N: Newton; NURBS: Non uniform rational b-splines; PCL: Posterior cruciate ligament; PCLa: Posterior cruciate ligament anterior bundle; PCLp: posterior cruciate ligament posterior bundle; PE: Polyethylene; TKA: Total knee arthroplasty.

## Competing interests

The authors declare no conflict of interest with the content of this study.

## Authors’ contributions

Author AS acquired funding and designed the study. He performed virtual implantation of prosthesis in the FE-knee model and drafted the manuscript. Author MW developed the FE-model of the knee, conducted the solving process of the different FE-models, has done post processing, interpreted the results and participated substantially in drafting the manuscript. Author AS and MW contributed equally to this study. Author PW participated in designing the study and accompanied the proceeding of the study. Author PEM participated in the design of the study and helped to draft the manuscript. Author VJ conceived the study and participated in its design and coordination. Author CS participated in the designing of the study, developing the FE-Model of the knee with author MW, interpreted the results and participated in drafting the manuscript. All authors read and approved the final manuscript.
